# Immune Infiltration Landscape in Clear Cell Renal Cell Carcinoma Implications

**DOI:** 10.3389/fonc.2020.491621

**Published:** 2021-02-16

**Authors:** Yongfeng Wang, Ci Yin, Lele Geng, Weiyang Cai

**Affiliations:** ^1^ Department of Gynecology and Obstetrics, Yangpu Hospital, Tongji University School of Medicine, Shanghai, China; ^2^ Department of Neonatology, Shaanxi Provincial People’s Hospital, Xi’an, China; ^3^ Department of Plastic Surgery, Shanghai Ninth People’s Hospital, Shanghai Jiaotong University School of Medicine, Shanghai, China; ^4^ Department of Gastroenterology, The First Affiliated Hospital of Wenzhou Medical University, Wenzhou, China

**Keywords:** clear cell renal cell carcinoma, tumor infiltrating immune cells, Tumor Microenvironment, CIBERSORT, TCGA

## Abstract

The malignant phenotypes of cancer are defined not only by its intrinsic tumor cells but also by the tumor infiltrating immune cells (TIICs) recruited to the cancer microenvironment. Clear cell renal cell carcinoma (ccRCC) immune microenvironment plays an important role in the tumorigenesis. This research investigated the characteristics of immune cell invasion of renal cell carcinoma and provided clues for future clinical implementation. Retrospectively, ccRCC gene expression was analyzed with appropriate clinicopathological data from the Cancer Genome Atlas (TCGA) and GEO database up to December 2019. The CIBERSORT algorithm, meta-analysis, principal component analysis (PCA), Single-Sample Gene Set Enrichment Analysis (ssGSEA) and hierarchical agglomerative clustering were used to measure and evaluate the respective proportions of 22 cell types of immune infiltration using normalized gene expression data. We also focused on evaluating the association with TIICs subpopulations and clinical features and molecular subtypes. TIICs subpopulation, especially Macrophages subgroup, T follicular helper (Tfh) cells and CD8 T cells, all contribute to tumorigenesis. Unsupervised clustering analysis revealed that there existed two distinct TIICs subgroups with different survival patterns. TIICs are extensively involved in the pathogenesis and development of the ccRCC. Characterizing the composition of TIICs influences the metabolism of tumors, activity, level, stage, and survival of patients. Collectively, the TIIC analysis has the potential to assist in the assessment and selection of ccRCC prognosis and treatment.

## Introduction

Clear cell renal cell carcinoma (ccRCC) is the second most common tumor in urology. As one of the popular urinary tumors, the incidence rate increases by 2–4% per year (1). Surgery is the most important treatment method for most patients with RCC and has achieved good efficacy in early renal cancer patients. Nonetheless, recurrence and metastasis are the main factors influencing the survival of ccRCC patients. As is well known, the prognosis varied evidently among ccRCC patients with the same TNM stage due to the heterogeneous subgroup of cancer cells. The malignant phenotypes of cancers are defined not only by the intrinsic activities of tumor cells but also by the immune cells recruited to and activated in the tumor-related microenvironment (2).

ccRCC is a tumor type with high immunogenicity, and its tumor cells produce an immunosuppressive environment through several immunosuppressive mechanisms, such as disrupting active antigen presentation, reducing the impact of T cells, through immunosuppressive sensitivity, and the T-cell “inability” pathway. Notably, ccRCC occurrence and development are closely related to its immune microenvironment (3–6). ccRCC has been proven surrounded by a large number of inflammatory cells such as T cells, NK cells, macrophages, which can destroy the immune system of the patient through the abnormal transformation of dendritic cells (7). As is well known, clear cell RCC (ccRCC) has the highest T cell infiltration score among the 19 cancer types, and often overexpress Programmed cell death protein 1 (PD-1) ligand, programmed cell death ligand 1 (PD-L1) (8). A clinical trial showed that the PD-L1 inhibitor avelumab and the PD-1 inhibitor pembrolizumab plus axinib with that of sunitinib had better advantages on RCC compared to sunitinib (9). Other potential therapeutic immune checkpoints such as CD73, B7H3, TIGIT, TIM3, LAG3, CD39, and adenosine A2A receptors are also being investigated (10). But the efficiency of ccRCC immune therapy is still low, only few patients have a complete response to immunotherapy. For example, Nivolumab is a monoclonal antibody against PD-1, which has a good response rate in clinical trials. Despite these advances, more than 55% RCC patients become resistant to different immunotherapies. However, between the first phase (29%) and the second phase (21%), the decrease of the objective response rate indicates that the clinical efficacy of nivolumab may be limited to few ccRCC patients (11). The use of salvage nivolumab-ipilimumab in metastasis ccRCC after anti-PD-1/PD-L1 only received limited activity with a 10% response rate, and part of patients developing grade ≥ 3 immune-related adverse events (11). The lack of predicted biomarkers, gene mutations, cellular senescence and the immune tolerance are the main characteristic reasons for this dilemma (12–14). A recent study showed that ccRCCs have the largest number of pan-cancer-based indel mutations, which can produce a higher amount of neoantigens and cause robust adaptive immune response (15). These data have highlighted the theme of correctly identifying tumor immune environment, which are characterized not only by high immune cell infiltration but also the tumor-related immune response. In consideration of the complex circumstance, it is urgent to provide an in-depth overview of ccRCC immune microenvironment, which sheds light on the immune regulatory mechanism.

To better understand the intricate relationship between the immune system and the tumor microenvironment, it is helpful to characterize each tumor-infiltration immune cell population to illustrate the breadth of tumor-specific functions. Here, we addressed this tissue by measuring qualitative features of the TIICs based on the deconvolution algorithm and verified the outcomes by immunohistochemistry and meta-analysis. We also set out to evaluate and dissect the prognosis of tumors with the identified cells to add a new way of clinical diagnosis, which leads to identifying the relationship between the composition of the TIICs and immunoreactions, molecular subtypes, and the TMN stage. It is hoped that this immune landscape could provide a more accurate understanding of ccRCC development and anti-tumor immunotherapy.

## Materials and Methods

### Data Acquisition

We retrospectively selected RCC gene expression with relevant clinicopathological data from the TCGA (https://cancergenome.nih.gov/) and GEO database (https://www.ncbi.nlm.nih.gov/geo/) until December 2019. We searched for the term “renal cancer” and “Homo sapiens” to select the appropriate datasets. The general working algorithm for GEO data was summarized in **Figure S1**. All research with data on gene expression (containing at least 20 samples) from primary human ccRCC is considered eligible, with no unique exclusion criteria being applied. The study used 19 cohorts of samples from patients with RCC for this study: GSE781, GSE46699, GSE53000, GSE6344, GSE53757, GSE11151, GSE68417, GSE15641, GSE16449, GSE16441, GSE105261, GSE36895, GSE40911, GSE40435, GSE85285, GSE73731, GSE79449, GSE68748, and TCGA-KIRC. Chips were summarized, together with accession numbers, in **Table 1**. In total, 2058 ccRCC cases were acceptable for subsequent analysis. Each tumor sample corresponded to one ccRCC patient. The RNA-sequencing data were processed *via* R limma package, setting *P*≤ 0.01, fold change ≥ 1.5 as the cutoff line.

**Table 1:** The detailed information of every RCC chips.

Accession	Platform	Number of normal samples	Number of cancer samples
**GSE781**	GPL80	10	20
**GSE6344**	GPL96	20	20
**GSE11151**	GPL6947	3	28
**GSE36895**	GPL96	23	28
**GSE40435**	GPL96	101	101
**GSE46699**	GPL570	65	65
**GSE53000**	GPL570	3	56
**GSE53757**	GPL15207	72	72
**GSE68417**	GPL6947	14	29
**GSE16449**	GPL6947	18	52
**GSE105261**	GPL6947	9	35
**GSE15641**	GPL17077	23	49
**GSE16441**	GPL14500	9	35
**GSE40911**	GPL3985	18	26
**GSE68784**	GPL20187	0	74
**GSE85258**	GPL570	15	15
**GSE73731**	GPL570	0	265
**GSE79449**	GPL9250	0	74

**Figure 1:**
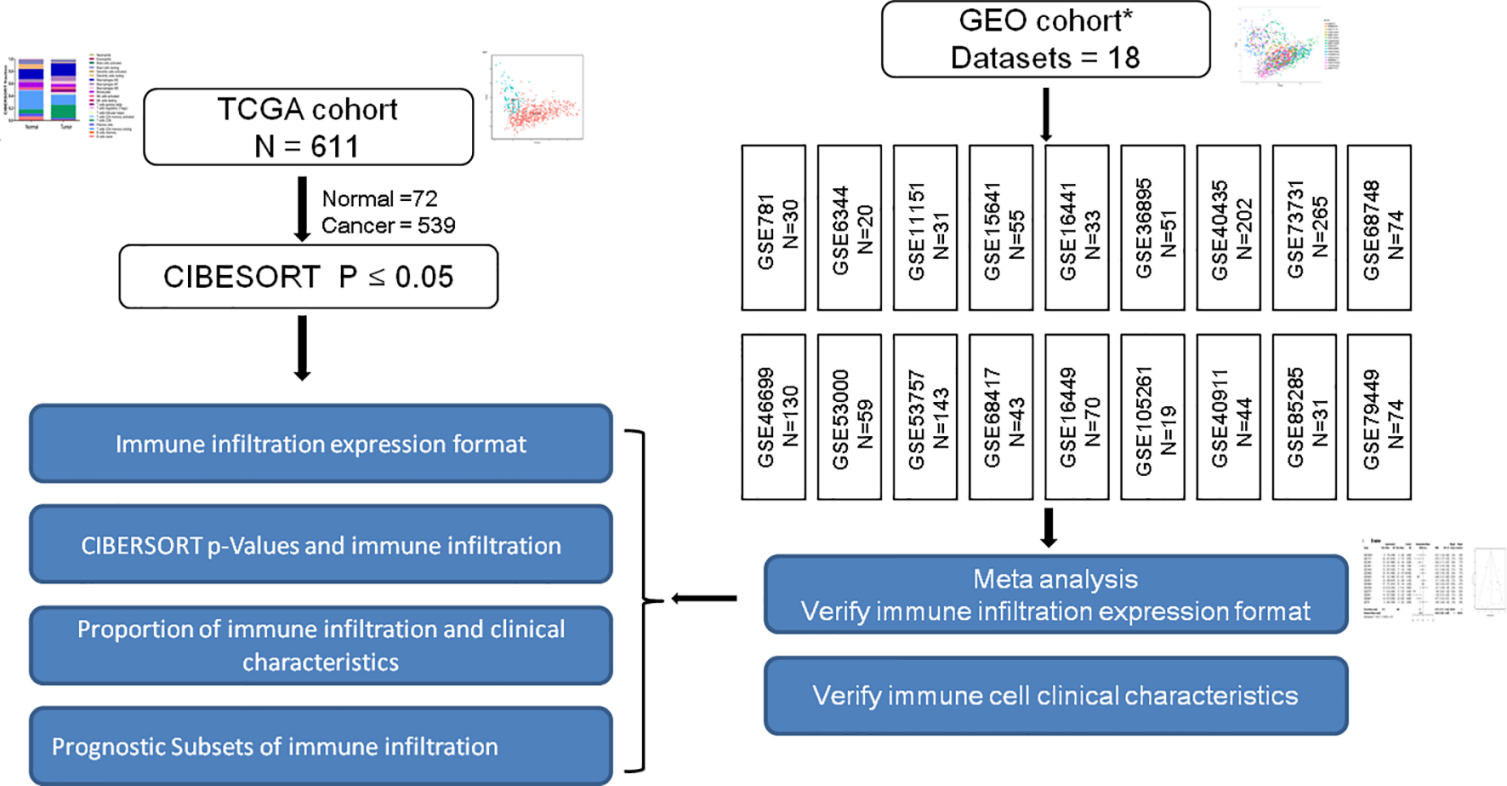
Is a representation of the flowchart of the overall study design. RCC gene expression data retrospectively selected from TCGA and GEO database up until June 2019. The constituent pattern of the immune microenvironment of RCC was systematically described and the differences between diverse immune clusters and gene subtypes were explored. Immune infiltration expression and clinical characteristics in GEO cohorts were verified. * **Figure S1** shows the general work algorithm of the GEO data.

Affymetrix SNP 6.0 arrays data of TCGA-KIRC were downloaded from TCGA (https://cancergenome.nih.gov/). The SNP profiling was determined according to the set of discrete copy number calls provided by GISTIC: deep loss/homozygous deletion (−2), shallow loss/hemizygous deletion (−1), low-level gain (1), and high-level amplification (16). Then somatic mutations and copy number variations were compared between normal and RCC tissues. Subsequently, TCGA-KIRC, GSE40435, GSE40912, GSE73731, GSE79449, and GSE105261 expression profiles and their corresponding clinical data were manually organized for prognosis and molecular subtypes analysis. Description of the layout of the analysis and the samples selected were shown in detail in the flow chart **Figure 1**.

### Inference of Immune Cell Infiltration

Normalized gene expression data were used to measure the relative proportions of 22 types of infiltrating immune cells using the CIBERSORT algorithm based on a set of reference gene expression data to infer different immune cell type proportions (17, 18). CIBERSORT P-value represented the confidence of the results of each test. We also removed this gene class at random in increments from 10% to 10% of the genes remaining. Such results indicated that the precision of the inferred immune cell proportions was correct, while the factor was limited to cases with an inferred immune cell count CIBERSORT *P*≤ 0.05 and barcode genes >40% (**Supplementary Figure 2**). The geometric mean of GZMA and PRF1 was calculated as an immune cytolytic activity, which represents another measure of immune infiltration (19).

### Meta-Analysis

The meta-analysis was performed by Review Manager 5.3 to adhere to the expression layout of each immune cell infiltrate in the ccRCC. Continuous outcomes were estimated as a standard mean difference (SMD) with a 95% confidence interval (CI). The DerSimonian and Laird method was referred to as the standard method, which offered an average impact estimate of the heterogeneity of effects across a series of chips. *P* < 0.05 was statistically significant. We also studied and analyzed in depth the composition of TIICs in the ccRCC to implement more scientific conclusions.

### Immunohistochemistry Data

The Immunohistochemistry results were obtained from the Human Protein Atlas (HPA, https://www.proteinatlas.org/) database. The immunohistochemistry results were used to compare the protein levels of selected genes.

### Principal Component Analysis

Principal component analysis (PCA) involves a mathematical procedure that transforms several (possibly) correlated variables into a (smaller) number of non-correlated variables called principal components. The eigenvector associated with the largest eigenvalue has the same path as the first principal component. As a result, this study examined the group-bias datasets and the individual differences by PCA, thus proving the credibility of the results in-depth.

### RT-PCR

Total RNA was used for cDNA synthesis with the help of a RT reagent kit (Takara, China). Each cDNA was amplified using the SYBR green (Takara) with the ABI Prism 7300 Real-time PCR system (Applied Biosystems, USA). The following primers were used: for human IDO1, 5′ -CCCACA CTGAGCACGGACGG-3′ and 5′ -TTGCGGGGCAGCACCTTT CG-3′; for human IDO2, 5′ -CAATCCAGCCATGCCTGTGGGG-3′ and 5′ - TGGGCTGCACTTCCTCCAGAGT-3′; for human LAG3, 5′-TCTCTCAGGCCTCCGACTGGTCATTTTG-3′ and 5′-TCCTGCAGATGGATATGGAGGTGTAGGTC-3′; for human IFNG, 5′ -CAGGTCATTCAGATGTAGCCGGAT-3′ and 5′ -TCATGTATTGCTTTGCGTTGG -3′; for human CTLA4, 5′ -GGATTTCAGGGGCACAAGC -3′ and 5′ -CCTGGAGATGCATACTACCACACA -3′.

### Single-Sample Gene Set Enrichment Analysis

The enrichment scores of the immune term were quantified by single-sample gene set enrichment analysis (ssGSEA) in the R package gsva (20). The specific markers of each immune gene set were listed in **Table S1**. Checkpoint score was defined as the average of the standardized values of IDO2, TIM-3, IDO2, PDL-1, CTLA4, LAG3, and TIGIT. Pro-inflammatory cytokines point included marker genes IFNG, IL-1A, IL-1B, and IL-2. Anti-inflammatory cytokines included IL-4, IL-10, IL-11, and TGFB1 (21). The correlation between the composition of the TIICs and the immune score was calculated using the Pearson correlation.

### Hierarchical Clustering Analysis

Samples with qualitatively different immune cell infiltration patterns were grouped using hierarchical agglomerative clustering. A combination of the Elbow method and the Gap statistic were used to explore the likely number of distinct immune clusters in the data. This approach assigned each sample into a data frame, finding the closest (most similar) cluster pair and combining it into a single cluster. Two clusters are found in ccRCC patients in the consensus matrix heatmap. Values were rescaled to be between zero (for the smallest observed value) and one (for the highest observed value) for each cell type to ensure comparability between the rare (low overall proportion) and abundant (high overall proportion) cell types. The associations between clusters and clinical outcomes were tested using a log-rank test. Next, the study analyzed the survival effect of each composition of the TIICs in detail.

### Statistical Analyses

Patients with CIBERSORT *P*-value of ≤ 0.05 were included in the following survival analysis. The associations of immune cell infiltrate and corresponding overall survival (OS) were analyzed by log-rank test. Analyses were also conducted separately for the VHL wildtype and mutant, which elucidate the fundamental difference in the most remarkable RCC mutant gene subgroup. All results were shown by the forest plot. For survival analysis, the quartile of each cell type was calculated and modeled as a continuous variable for more interpretable hazard ratios (HRs). The Chi-square test was performed to investigate the relationship between tumors and immune cell infiltration. All analyses were performed using R version 3.3.2. All statistical tests were two-sided and P-value ≤0.05 was considered statistically significant.

## Results

### The Landscape of Characterizing Tumor Infiltrating Immune Cells Composition in Clear Cell Renal Cell Carcinoma

**Figure 2:**
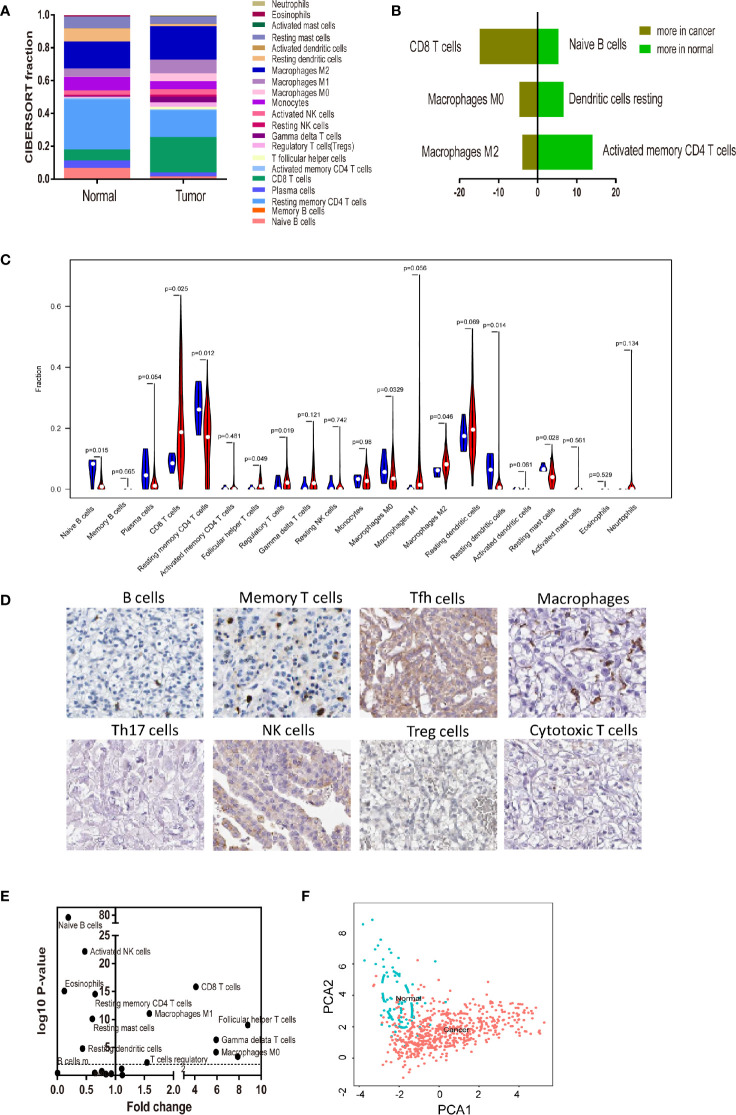
Landscape of microenvironment TIICs composition in RCC. **(A)** Relative microenvironment TIICs proportions in normal and RCC tissues. **(B)** Quantified changes of infiltrating immune cell composition in normal and RCC tissues. **(C)** Violin plot of the proportions of TIICs subpopulation. (blue represents normal tissue, red represents RCC). **(D)** Immunohistochemical stainings showed that the expression trends of different levels of specific immune cells. **(E)** Volcano Plot visualizing the differentially infiltrated immune cells. **(F)** Principal components analysis (PCA) analyzed all RCC samples. The proportions of immune cells from normal and RCC displayed distinct group- bias clustering and individual differences by PCA.

To systematically describe the constitutive pattern of the immune cell proportions of the microenvironment, we first used CIBERSORT to calculate the immune cell composition of ccRCC tissues. CIBERSORT is a gene expression-based deconvolution algorithm that allows for highly sensitive and specific discrimination of human tissue. As shown in **Figure 2A**, the distribution of the TIIC composition of the tumor tissue was completely different from that of normal tissue. In particular (**Figure 2B**), resting memory CD4 T cells and resting dendritic cells in healthy renal tissue were most common; whether CD8 T cells and macrophages were most frequent in tumor tissue. When compared to paracancerous tissue, the proportions of CD8 T cells, Tfh cells, regulatory T cells (Tregs), M1, and M0 macrophages were significantly increased, whereas the proportions of naive B cells, resting memory CD4 T cells, Monocytes, resting dendritic cells and resting mast cells were relatively lower (**Figure 2C**). Immunohistochemical (IHC) stainings showed that the expression trends of different levels of specific immune cells (**Figure 2D**). Convincingly, the proportions of normal and ccRCC immune cells showed distinct clustering of biases and individual PCA differences (**Figures 2E, F**).

### Meta-Analyze Validates the Proportions of Tumor Infiltrating Immune Cells in the Clear Cell Renal Cell Carcinoma

**Figure 3:**
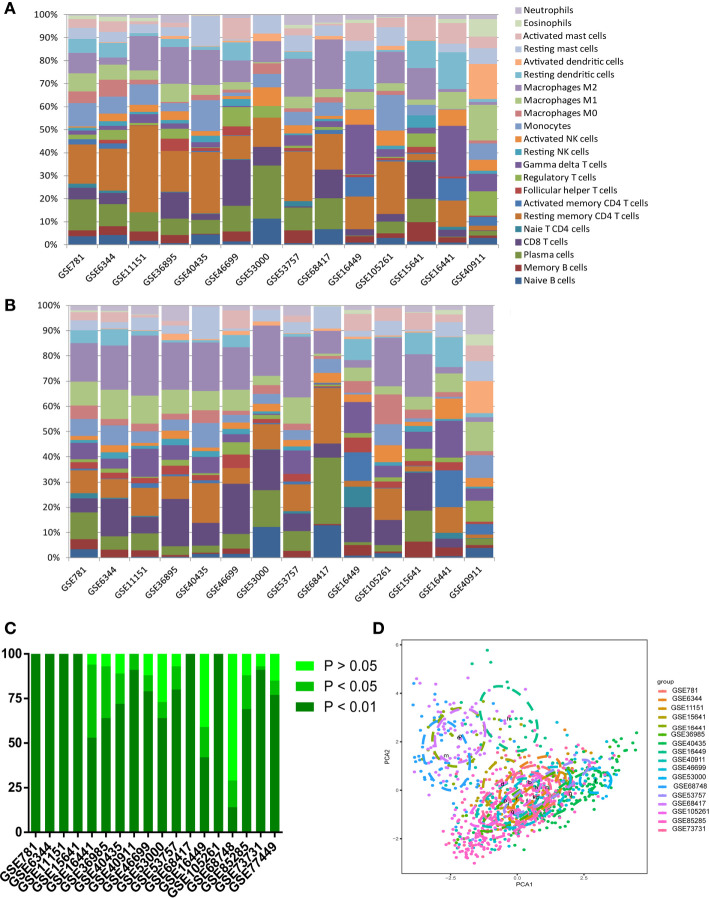
Summary of GEO inferred GEO TIICs composition. **(A)** Bar charts summarize GEO chips concrete immune cell subset proportions of normal renal tissues. **(B)** Bar charts summarize GEO chips concrete immune cell subset proportions of RCC tissues. **(C)** The proportion of chips with different P-value thresholds. **(D)** PAC depicts the variation of 22 TIICs subpopulations cross all sample from GEO datasets.

To confirm the accuracy of the results of this study, the researchers inferred its accuracy in other independent ccRCC datasets both containing rental tumor and adjacent normal specimens. The researchers checked the ccRCC microarray in the GEO dataset and obtained 15 qualifying microarrays. **Table S2** summarizes all studies and related platforms maximum of 403 normal and 1044 ccRCC tissues. Bar charts summarize TIICs subpopulations of normal tissues (**Figure 3A**), ccRCC (**Figure 3B**), and CIBERSORT *P*-value (**Figure 3C**). Although the above chip profiles were obtained from different specimen sources and platforms, the proportions of the TIIC subpopulation did not show evidence of cohort bias (**Figure 3D**).

**Figure 4:**
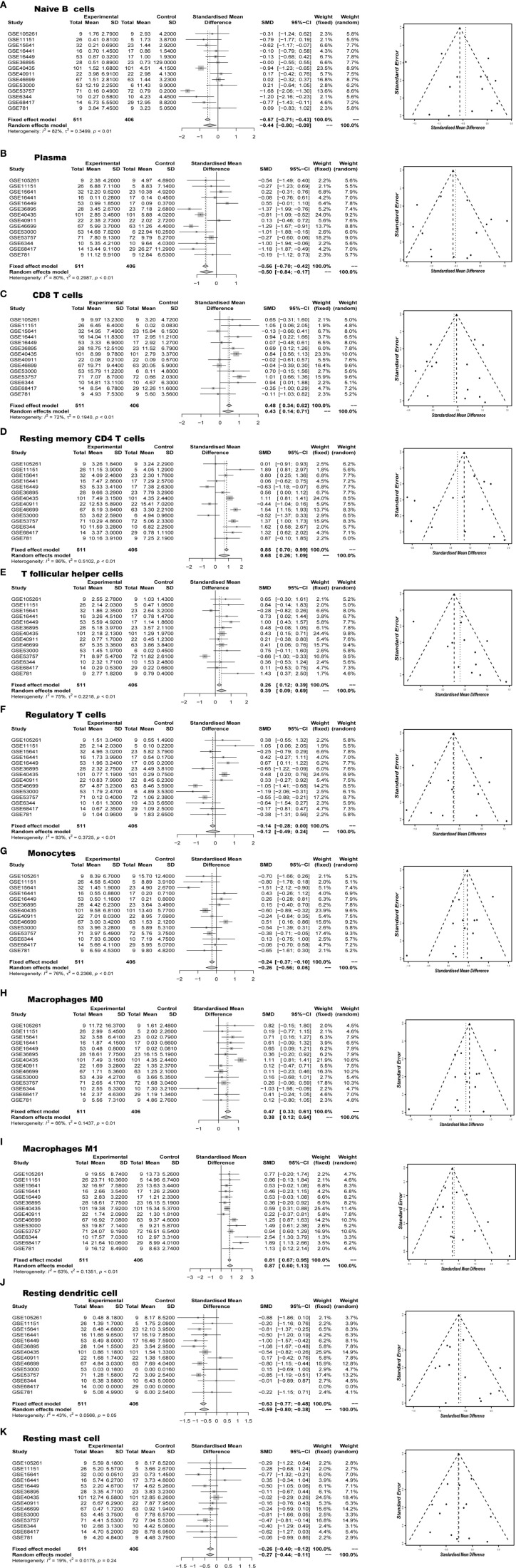
Meta-analyze verified aberrant TIICs composition **(A)** Naïve B cells, **(B)** Plasma, **(C)** CD8 T cells, **(D)** Resting memory CD4 T cells, **(E)** T follicular helper cells, **(F)** Regulatory T cells, **(G)** Monocytes, **(H)** Macrophages M0, **(I)** Macrophages M1, **(J)** Resting dendritic cells, **(K)** Resting mast cells in RCC.

Next, we performed a meta-analysis of each distinct composition of TIICs in the ccRCC. **Figures 4A–K** showed different meta-analyze composition of TIICs and its corresponding heterogeneity test Obviously, naive B cells (95% CI, −0.8 to -0.09; *P* < 0.01), plasma cells (95% CI, −0.84 to -0.17; *P* < 0.01), monocytes (95% CI, −0.56 to 0.05; *P* < 0.01), resting dendritic cells (95% CI, −0.80 to -0.38; *P*= 0.05) and resting mast cells (95% CI, −0.44 to -0.11; *P*= 0.24) exhibited a decrease tendency; whereas CD8 T cells (95% CI, 0.14 to 0.71; *P* < 0.01), resting memory CD4 T cells (95% CI, 0.26 to 1.09; *P* < 0.01), M0 macrophages (95% CI, 0.12 to 0.64; *P* < 0.01), and M1 macrophages (95% CI, 0.60 to 1.13; *P* < 0.01) exhibited an increasing tendency in ccRCC tissues. Although the above genomic profiles were collected using a variety of techniques and specimen sources, these samples did not show cohort bias and confusing bias. In brief, these meta-analyzing findings, together with previous studies, suggested that the result from this study were strong enough to discriminate against the subpopulation of TIICs in the ccRCC.

### The CIBERSORT *P*-Value Reflects the Overall Proportion of Immune Cells

**Figure 5:**
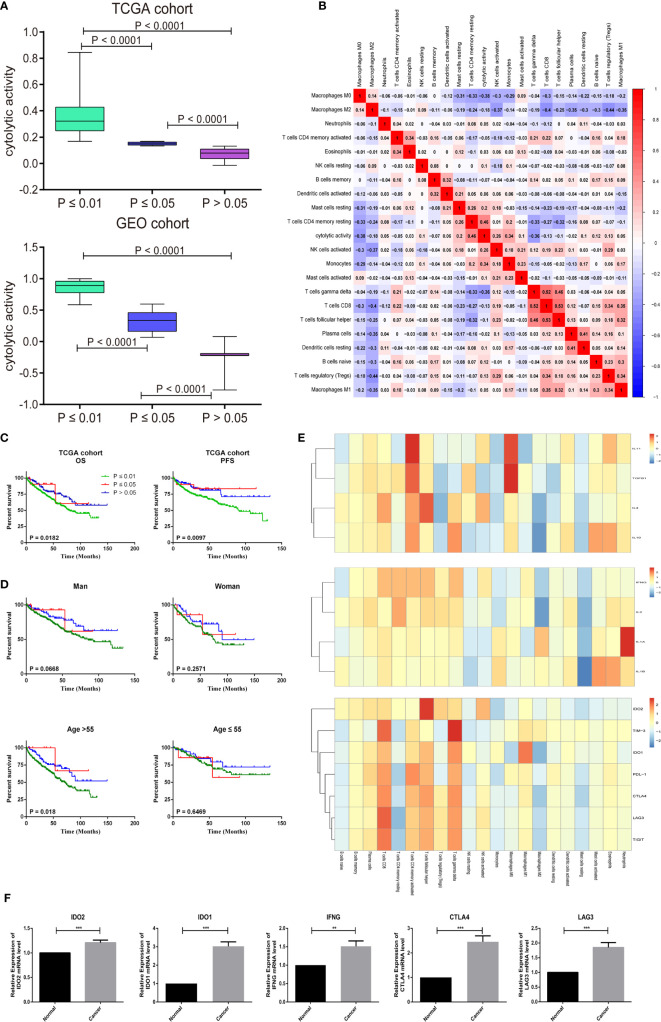
CIBERSORT *P* -values reflect the immune cytolytic activity. **(A)** Box plots describe the association between immune cytolytic activity and CIBERSORT *P*-value **(B)** Correlation matrix of all 22 immune cell proportions and immune cytolytic activity in the TCGA cohort. **(C)** Survival plots of groups are defined by CIBERSORT *P*-value separately. (OS, overall survival; PFS, progression-free survival) **(D)** Effect of overall immune infiltration on overall- survival man or woman in patients; Effect of overall immune infiltration on overall- survival in patients ≤55 or >55 year old. **(E)** Correlation matrix of all 22 immune cell proportions and pro-inflammatory cytokines (IFNG, IL-1A, IL-1B, and IL-2), anti-inflammatory cytokines (IL-4, IL-10, IL-11, and TGFB1), and checkpoint recognition (IDO2, TIM-3, IDO1, PDL-1, CTLA4, LAG3, and TIGIT). **(F)** The human paired normal renal and ccRCC tissue IDO2, IDO1, IFNG, CTLA4 and LAG3 mRNA level was measured by reverse-transcription PCR. Data represent mean standard deviation.

The CIBERSORT *P*-Value reflects the ratio of immune cells to non-immune cells, and the larger the proportion of immune cells, the smaller the corresponding *P*-value. We then checked the correlation between the P-value of CIBERSORT and the immune cytolytic activity. The metric of immune cytolytic activity was based on the geometric mean values of GZMA and PRF1. In the GEO and TCGA data sets, there is usually a close relationship between different P-value and cytolytic activity thresholds (**Figure 5A**, *P* < 0.001). Resting memory CD4 T cells and macrophage M0 were most strongly correlated with cytolytic activity (**Figure 5B**). Considering the important role of the composition of the TIICs in the prognosis, we further explored their clinical significance. The researchers found that patients with *P <*0.01 were more closely associated with deteriorating overall survival and PFS (**Figure 5C**). High infiltration of immune cells may also worsen survival in patients < 55 years of age (**Figure 5D**).

Frequent cancer immune signature processes usually included checkpoint recognition, IFN response, MHC class, APC recognition, HLA expression, inflammation promotion, and parainflammation. Next, we obtained a set of genes of the relative immune system from KEGG and used ssGSEA for the gene set to quantify these immune signatures (22). Surprisingly, we found that immune cells were very involved in the checkpoint reaction and various inflammatory reactions in the ccRCC. Therefore, the researchers explored in detail the relationship between TIICs and pro-inflammatory cytokines (IFNG, IL-1A, IL-1B, and IL-2), anti-inflammatory cytokines (IL-4, IL-10, IL-11, and TGFB1), and checkpoint recognition (IDO2, TIM-3, IDO1, PDL-1, CTLA4, LAG3, and TIGIT). As shown in the **Figure 5E**, CD8 T cells, Tfh cells, activated memory CD4 T cells, and gamma delta T cells had a close positive association with checkpoint recognition molecule. Activated memory CD4 T cells, Macrophages subgroup play a significant function in inflammatory cytokines response. To evaluate the mRNA expression level of key checkpoint recognition molecular IDO2, IDO1, IFNG, CTLA4, and LAG3 in ccRCC, we investigated the expression in 5 paired human ccRCCs and matching adjacent nontumor renal tissues. Not surprisingly, their mRNA expression levels are all significantly increased in tumors, which were in consistent with the above analysis (**Figure 5F**).

### Proportional Distribution of Immune Cells and Clinical Characteristics

CIBERSORT P-value also represented the confidence of the results of each sample. In increments of 10%, we randomly deleted this gene group until 10% of the genes remained. These findings suggested that the accuracy of the inferred immune cell proportions was accurate while variable limited to cases with CIBERSORT P ≤ 0.05 and barcode genes >0%. Thus, all of the follow-up analysis was conducted for samples with CIBERSORT *P* ≤ 0.05 (**Supplementary Figure 2**). We combined medical characteristics with the composition of TIICs and further investigated whether the TIIC subpopulation was statistically associated with ccRCC development. As shown in **Supplementary Figure 3A**, the proportion of Macrophages M1, Tregs, CD8 T cells, Tfh cells, and activated memory CD4 T cells were increasing accompany with advanced tumor T stage; whereas resting memory CD4 T cells, resting dendritic cells, and resting NK cells were down-regulated. For the M stage, mast cells, and memory CD4 T cells were all associated with ccRCC metastasis (**Supplementary Figure 3B**). Dendritic cells, mast cells, T cells CD4, Tregs, Tfh cells, and Macrophages also function in diagnosing ccRCC pathological stage and grade (**Supplementary Figures 4A, B**). To further confirm the clinical value of TIICs, the validation cohort was used in the GEO datasets provided with clinical information. As shown in **Supplementary Figures 5**–**9**, there was a clear statistical difference or similar pattern of change in the progression of the ccRCC. In summary, the analysis of the composition of the TIICs is very much part of the pathogenesis and development of the ccRCC.

### Immune Clusters Associated With Prognosis and Molecular Subtypes

**Figure 6:**
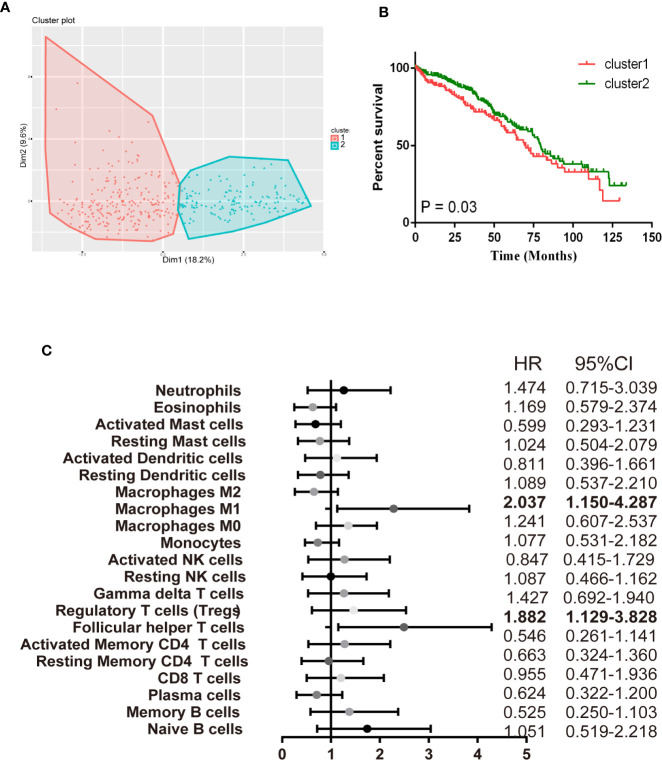
Immune clusters associated with RCC prognosis. **(A)** The scatter diagram shows the variation of the principal components of each patient, according to the cohort to which they belong. **(B)** Kaplan-Meier survival analysis of patients within different clusters. **(C)** Prognostic associations of TIICs subpopulation. Unadjusted HRs (boxes) and 95% confidence intervals (horizontal lines) are limited to cases with CIBERSORT *P*-value < 0.05.

Afterward, we assessed whether these selected TIICs subsets made sense to ccRCC patients as independent indicators. There were 405 ccRCC cases with a median follow-up of 43.74 months by restricting cases with CIBERSORT *P* < 0.05. Detailed results are shown in **Table S2**. The variation of TIICs subsets played a significant role in tumor progression, thus we firstly performed a hierarchical clustering study of 22 TIICs subpopulations for each patient. The optimal number of clusters was determined by combining the Elbow method with the Gap statistic method. The scatter diagram revealed that 2 clusters were identified as individualized clusters (**Figure 6A**). Cluster1 was defined by a relatively high level of plasma cells, resting memory CD4 T cells and resting NK cells, fewer CD8 T cells and Tfh cells. Specific immune clusters are associated with specific survival patterns (**Figure 6B**). The researchers further elaborated on each TIICs subpopulation correlation with ccRCC survival progress by univariate Cox regression analysis. **Figure 6C** shows the unadjusted HRs and the 95% confidence intervals for quartile proportions per cell type. Tfh cells (OR = 2.037, 95%CI = 1.150–4.287; *P* = 0.003), M1 (OR = 1.882, 95%CI = 1.129–3.828; *P* = 0.021) were significantly associated with poor overall survival. In summary, these findings suggest that TIICs subpopulations, especially the Macrophages subgroup, Tfh cells, and CD8 T cells, all contribute to tumorigenesis. These findings support the use of these tumor-infiltration lymphocytes as predictors of ccRCC progression.

### Identification of Prognostic Subsets of Tumor Infiltrating Immune Cells in Clear Cell Renal Cell Carcinoma

**Figure 7:**
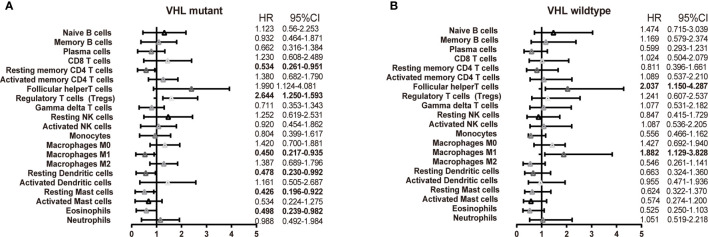
Subgroup overall survival analyses of VHL mutant. The prognostic effect of 22 immune cell subsets by VHL mutant **(A)** and wild type **(B)**.

VHL gene protein can inhibit the hypoxia inducing factor (HIF) and function in inhibiting the tumor. Up to 92 percent of clear cell carcinoma patients were characteristic of VHL gene mutant, which leads to overproduction of VEGF and then contributes to angiogenesis of ccRCC. There existed a similar outcome in the TCGA dataset that VHL was the most remarkable SNP in ccRCC (**Supplementary Figure 10**). It is commonly accepted that VHL inactivation is a marker of ccRCC following target immunotherapy. Thus, it is urgent to investigate the interaction of the immune response with VHL molecular subtypes. We performed an exploratory subgroup analysis of the prognosis of the 22 immune cell subsets using molecular subtype defined by VHL mutant (**Figures 7A, B**). T follicular helper (Tfh) cells showed a strong association with poor outcome in the mutant (HR = 1.99, 95% CI 1.124–4.081) and wild type (HR = 2.037, 95% CI 1.150–4.087). Most strikingly, macrophages M1n have the opposite effect in VHL‐related patients, with the favorable outcome in the mutant subgroup (HR = 0.45, 95% CI 0.217–0.935) and unfavorable outcome in the wildtype subgroup (HR = 1.882, 95% CI 1.129–3.828). Collectively, such findings suggest that there is substantial variation in the process of immune penetration across the ccRCC—partly defined by the molecular characteristics of the primary tumor—and that this influences the clinical outcome.

## Discussion

Approximately 20% of tumor-relative deaths worldwide are associated with unresolved infections or chronic inflammation (23). The components and complicated interaction of tumor immune cells exert significant effects on the aggressiveness of malignant cells. Immune cells as key components of the tumor microenvironment, its checkpoints or adoptive cell transplantation have made great breakthroughs in effective response of various types of cancer, which contribute to improve the prediction of clinical outcome and progressively become an effective target of drugs. Recent trials of immune checkpoint drugs have proven that they can significantly prolong the survival of a subset of patients with solid tumors. For example, adoptive cell therapy (ACT) can mediate the regression of a variety of cancers, including melanoma, cervical cancer, lymphoma, leukemia, cholangiocarcinoma, and neuroblastoma. Immunotherapeutic PD-1 by pembrolizumab monotherapy has achieved significant improvement on the survival of solid tumor patients, such as non-small-cell lung carcinoma, gastric cancer, renal cell carcinoma, melanoma, cervical cancer, and triple-negative breast cancer (24–26).

A large body of evidence has proved the important impact of immune cells in determining the progression of the ccRCC pathogenesis. Experimental techniques such as flow cytometry and immunohistochemistry are usually used to measure the expression level of immune cells, but the accuracy and credibility are still low. Owing to technical limitations, these studies have necessarily been limited to a very narrow view of the immune response, ordinarily including only one or two cell types. Thus, a comprehensive systematic assessment of the immune landscape is urgently needed to make a more accurate understanding of ccRCC development, compared with traditional single-factor predictors. CIBERSORT, as a rising technology, has been implemented in the exploration of breast cancer, liver cancer, colorectal cancer, lung cancer, melanoma, and leukocyte. And we firstly performed CIBERSORT analysis of ccRCC, with the order to assess the mystical immune landscape. To our knowledge, we performed the most comprehensive analysis of the immune cell composition and its clinical effects. It is well recognized that there exists a marked infiltration of different types of immune cells, and the distribution, tissue localization, and cell types are significantly associated with the progression and survival of ccRCC. As shown in **Figures 2** and **4**, the proportions of CD8 T cells, macrophages M0, macrophages M2 were elevated in RCC, while B cell naïve, resting dendritic cells and resting memory CD4 T cells were declined. The same expression trend has been widely proven in ccRCC relative study. Laurence et al. concluded that there existed increased CD8 + T cells and the down-regulated CD4 + T cells in human ccRCC tissues (27). It reported that ccRCC cells have a better ability to absorb macrophages than normal renal epithelial cells (28).

As increasing evidence have revealed the clinical significance of immune infiltration in ccRCC development, thus, we also made a comprehensive analysis of the clinical impact of the immune cell, which may help clinicians predict ccRCC patient outcomes more reliably and precisely. We discovered that CD8 T cells, activated memory CD4 T cells, Tregs, Tfh cells, resting dendritic cells, macrophages M0 and macrophages M2 infiltration were associated with poor prognosis for ccRCC patients. In **Supplementary Figures 3**–**4**, we observe that CD8 T cells, activated memory CD4 T cells, Tregs, Tfh cells infiltrations consistent with tumor T&M, grade, and stage trend. Additionally, our data firstly provided a widespread view of the process of ccRCC immunity response. The tumorous immune responses are characteristic of specialized cell types interacting in a highly coordinated manner. Increasing understanding of the mechanisms of T cell recognition and function has led to a great development of novel immunotherapies for ccRCC. A subpopulation of T cells could further differentiation, such as CD8 T cells and Treg, influence ccRCC carcinogenesis process in multi-aspect. To better understand the innate character of the T cell immune response to renal tumors, it is necessary to enumerate sorts of T immune cells in a way that accounts for the breadth of their specialized functions. We first discovered that CD8 T cells, Tfh cells, activated memory CD4 T cells and gamma delta T cells have a close and positive relationship with checkpoint recognition molecule (**Figure 6**). Multiple feedback mechanisms exert in simulating T cells activation, regulating immune function, and preventing an excessive antigen response. For example, Tregs is a subgroup of T cells with negative immune regulation, mainly secreting IL-10 and TGF-β, which can inhibit the activation and proliferation of anti-tumor effector cells and are associated with tumor immune escape (29, 30). Moreover, Tregs can induce phenotypic and functional changes in T cells and dendritic cells, thereby promoting cell senescence and inhibiting anti-tumor effects in the tumor microenvironment (31). The expression of Tregs in peripheral blood and tumor tissues of ccRCC patients was proven mediated by immune chemotaxis, which accelerates cell senescence and other mechanisms to inhibit the activation and proliferation of anti-tumor effector cells and participate in tumor immune escape (32). Cytotoxic T-lymphocyte antigen-4 (CTLA-4) blockage weakens the function of Tregs, resulting in a stronger anti-tumor response. Inhibition of mTOR promotes the expansion of immunosuppressive Tregs that can strengthen the effect of anti-tumor immune responses in ccRCC.

Macrophage infiltration in ccRCC was enhanced and associated with prognosis (33). Macrophages have been to found influence the aggressiveness and prognosis of ccRCC and associated with poor outcome (34, 35). We get the same result in our analysis. Notably, M1 macrophages invasion is consistent with the ccRCC T&M trend, while the M0 was consistent with the ccRCC phase and grade trend. The differences between macrophage “polarized states” are subject to some controversies, but the two states both exist in the core of tumor (36). The data shows that M0 macrophages were associated with poor tumor prognosis, and M1 were associated with distant tumor metastasis, indicating prognostic significance (**Supplementary Figures 3**–**4** and **Figure 6**). Macrophages are more easily recruited into ccRCC tissues than surrounding non-tumor tissues, which may increase the invasion capacity of ccRCC cells. Infiltrating macrophages can induce epithelial-mesenchymal transformation by activating the AKT/mTOR signal, increasing the cancer stem cell-like population, leading to increased invasion of ccRCC cells (28). Macrophages may either exert pro-tumorigenic effects accelerating cancer development or anti-tumorigenic effects according to differentiation patterns into M1 or M2 subtypes (37). This is of exceeding importance because treatments to complete the tumor-promoting functions of macrophages are already in early phase clinical trials. For example, Arun k. Iyera et al. selectively delivered Sorafenib in combination with tumor hypoxia directed nanoparticle to hypoxic tumors, selectively down-regulated tumorigenic M2-macrophages, and up-regulated macrophages of tumor-killing M1 caused hypoxic necrosis of tumors (38).

VHL is a substrate recognition component of the E3 ligase complex associated with ubiquitinated hif-1 α and hif-2 α (39). VHL loss is the most common genetic characteristic of ccRCC, with 92% of ccRCC showing VHL mutant (40, 41). Inactivation of the VHL leads to overproduction of VEGF, which contributes to the angiogenesis of ccRCC. The absence of VHL exerts a significant effect on ccRCC progression and affecting the responsiveness of immune checkpoint therapy. Thus, it is urgent to investigate the interaction of the immune response with VHL molecular subtypes, which potentially provide implications for the inclusion of immune infiltrates as part of clinical prognostic models. The treatment for VHL substrate HIF, such as a vascular endothelial growth factor (VEGF) inhibitor, is currently the standard treatment for ccRCC. Furthermore, it is urgent to identify new and stable VHL substrates to propose new therapeutic regimens for ccRCC treatment. We analyzed the absent and wild-type of VHL in ccRCC and found that alterations of a variety of immune cells, including memory B cells, activated memory CD4 T cells, Tregs, M1 macrophages, M2 macrophages, and activated dendritic cells in two groups. In the absence of VHL group, we observed that M0 and M2 macrophages were positively correlated with the occurrence of ccRCC, while M1 was negatively correlated (41). This indicates variations in tumor microenvironment caused by VHL that lead to changes in ccRCC macrophage polarization, providing us with useful information to explore new checkpoints. VHL is related to hypoxia-inducible factors, and a large number of cytokines will be produced in the process of chronic hypoxia in tumors, which can change the utilization rate of oxygen, thus affecting the polarization of macrophages (33, 42). VHL-proficient ccRCC patients may be more sensitive to treatment with 9-cis-retinoic acid, which acts by regulating RXRα expression, compared with VHL-deficient ccRCC patients. These findings demonstrate a novel function of VHL, and highlight the potential of VHL expression as a therapeutic modality for the optimized treatment of ccRCC patients.

There are also some potential therapeutic agents such as Epacadostat, an inhibitor targeting indoleamine 2, 3-dioxidase (IDO) (43, 44), Galunisertib, which is an inhibitor of transforming growth factor-β (TGF-β) related with immune tolerance (45) and bms-986016, which is an inhibitor targeting t-cell function anti–lymphocyte-activated gene 3 (LAG-3) (46). Hence, the study also explored the relationship between TIICs and pro-inflammatory cytokines (IFNG, IL-1A, IL-1B, and IL-2), anti-inflammatory cytokines (IL-4, IL-10, IL-11, and TGFB1), and checkpoint recognition (IDO2, TIM-3, IDO1, PDL-1, CTLA4, LAG3, and TIGIT). The findings showed that CD8 T cells, Tfh cells, activated memory CD4 T cells and gamma delta T cells had a close positive association with checkpoint recognition molecule. Activated memory CD4 T cells and Macrophages subgroup play a significant function in inflammatory cytokines response. We believe that these pro-inflammatory cytokines and checkpoints can be explored as effective therapeutic targets.

Despite the momentous results obtained in this study, there still exist several shortcomings. Firstly and most important, although relative statistical methods involved in the analysis have been conducted to eliminate cohort bias, heterogeneity in these data still impede the repeatability at some level. Second, we only included microarray data in your analysis, there should be high-throughput sequencing data there. Third, CIBERSORT can only estimate the relative abundance of immune cells, which means some cell types may be overestimated or underestimated. Thus, if possible, further experiments could be performed *in vivo* and *in vitro* to verify these results.

In general, this study analyzed and revealed an important relationship between immune cell subsets and tumor type survival, further enhancing understanding of the clinical impact of immune responses, with particular emphasis on the different combinations of outcomes associated with different functional cell subsets, which provided potential targets for new drugs, helped to evaluate the prognosis, and supplemented the data for accurate cancer medicine to improve patient outcomes.

## Data Availability Statement

The datasets generated for this study can be found in the The Cancer Genome Atlas (TCGA) (https://portal.gdc.cancer.gov/) database: GSE781, GSE6344, GSE11151, GSE36895, GSE40435, GSE46699, GSE53000, GSE53757, GSE68417, GSE16449, GSE105261, GSE15641, GSE16441, GSE40911, GSE68784, GSE85258, SE73731, GSE79449.

## Author Contributions

WC and LG conceived and designed the experiments. YW and CY collected the data. WC analyzed the data. LG peformed the biochemical experiments. YW and LG wrote the manuscript. All authors contributed to the article and approved the submitted version.

## Funding

This work was supported by the Scientific Research Foundation of the First Affiliated Hospital of Wenzhou Medical University (grant no. FHY2019002)

## Conflict of Interest

The authors declare that the research was conducted in the absence of any commercial or financial relationships that could be construed as a potential conflict of interest.

## Supplementary Material

Supplementary Figure 1 General work algorithm of the GEO dataset

Supplementary Figure 2 Box plot of the distribution of CIBERSORT *P*-value and average Pearson’s correlation using datasets with progressively fewer (10% increments) barcode genes from the TCGA-KIRC cohort. The *P*-value was highly sensitive to the diminishing representation of the barcode genes.

Supplementary Figure 3 Association between the TIICs and clinicopathological features in RCC. **(A)** Infiltrating immune cells functioned in distinguishing the T stage. **(B)** Infiltrating immune cells functioned in distinguishing the M stage.

Supplementary Figure 4 Association between the TIICs and clinicopathological features in RCC. **(A)** Infiltrating immune cells functioned in distinguishing the pathological stage. **(B)** Infiltrating immune cells functioned in distinguishing grade.

Supplementary Figure 5 Validation clinical characteristics of the TIICs in GSE40912.

Supplementary Figure 6 Validation clinical characteristics of the TIICs in GSE79449.

Supplementary Figure 7 Validation clinical characteristics of the TIICs in GSE68748.

Supplementary Figure 8 Validation clinical characteristics of the TIICs in GSE73731.

Supplementary Figure 9 Validation clinical characteristics of the TIICs in GSE40435.

Supplementary Figure 10 Waterfall map depicts the top15 SNP in TCGA-KIRC.
