# Supplementary figures and images for: Immune Infiltration Landscape in Clear Cell Renal Cell Carcinoma Implications
Source: Front Oncol. 2021 Feb 16;10:491621. doi: 10.3389/fonc.2020.491621 (PMC7923891; doi:10.3389/fonc.2020.491621)

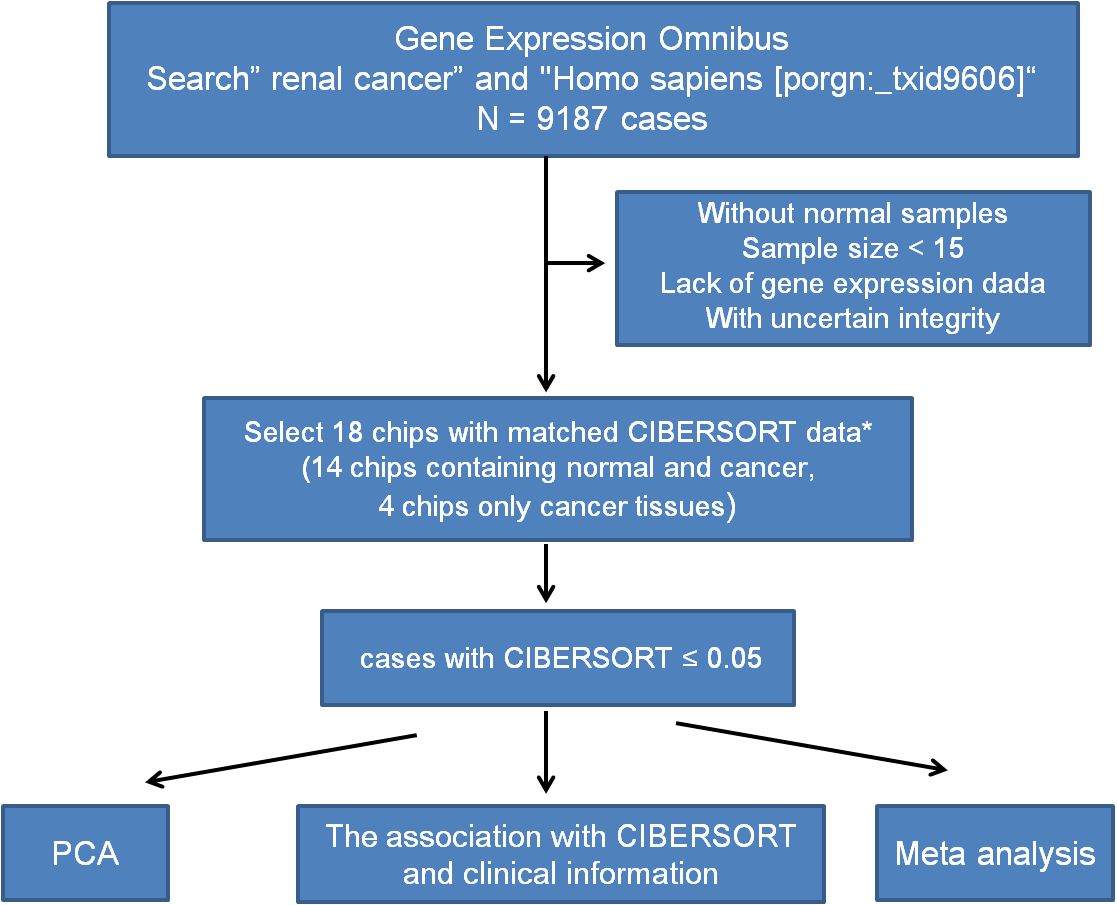

Supplement: Supplementary Figure 1 — General work algorithm of the GEO dataset [file Image_1.tif]

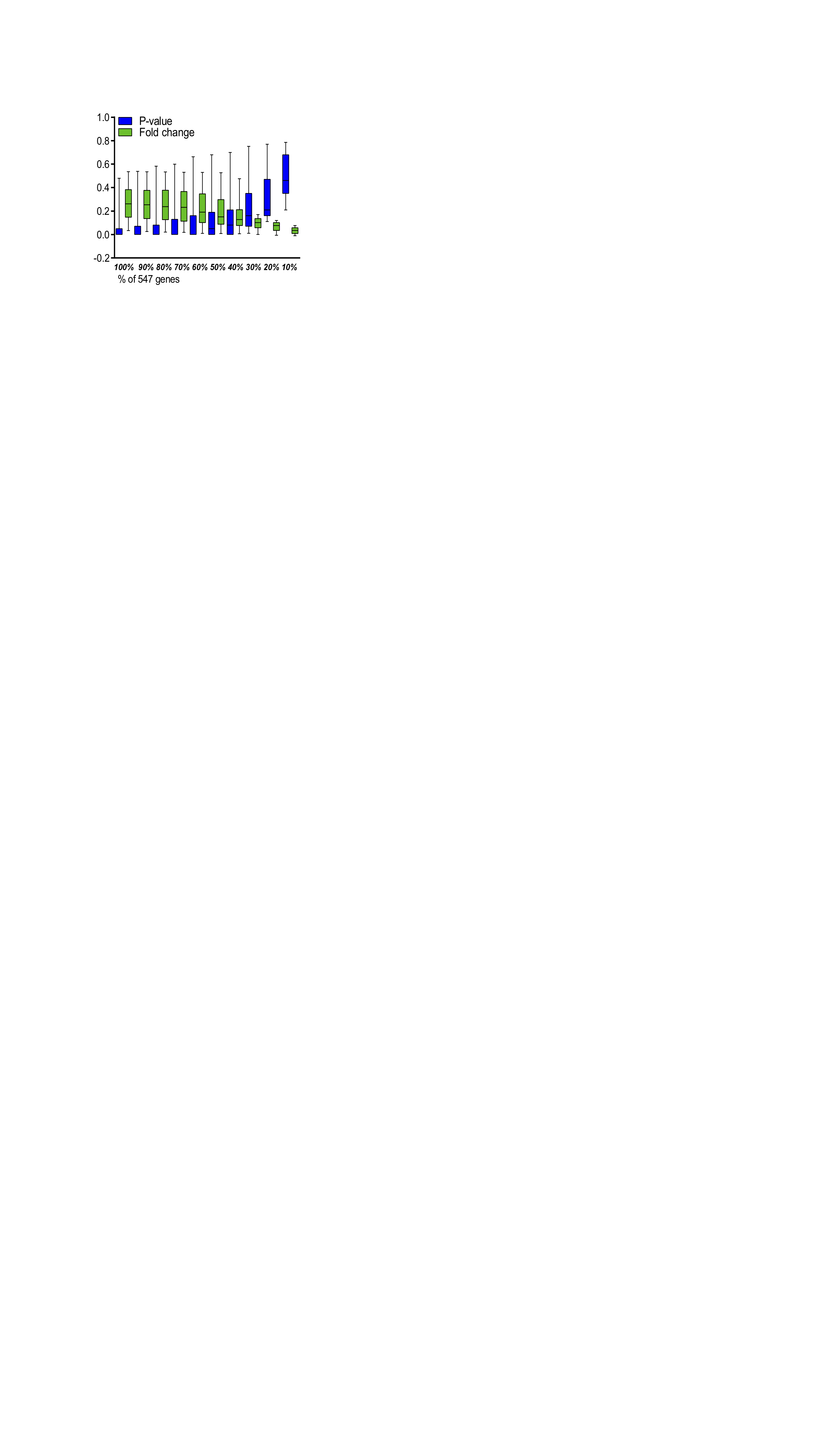

Supplement: Supplementary Figure 2 — Box plot of the distribution of CIBERSORT P-value and average Pearson’s correlation using datasets with progressively fewer (10% increments) barcode genes from the TCGA-KIRC cohort. The P-value was highly sensitive to the diminishing representation of the barcode genes. [file Image_2.tif]

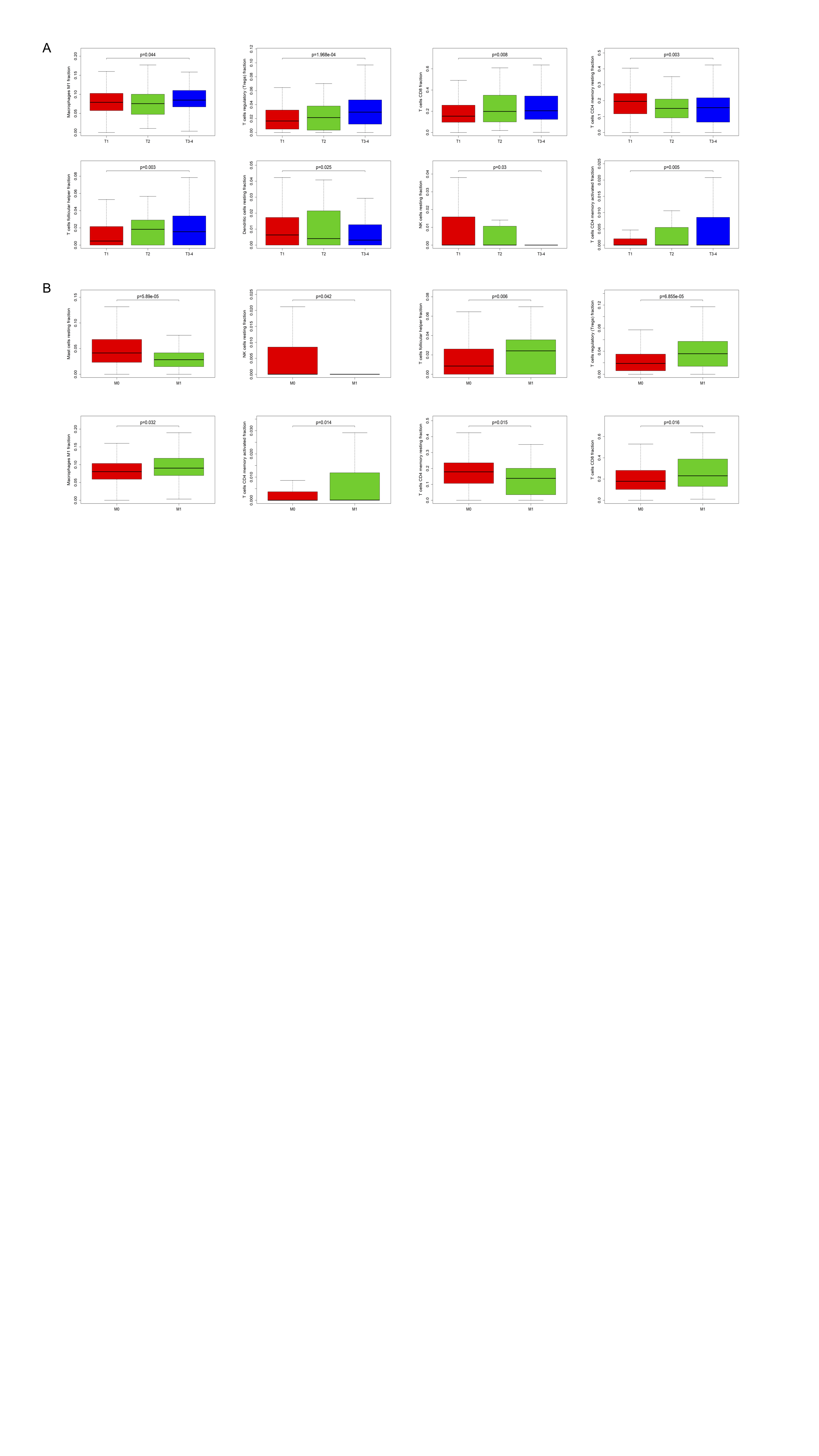

Supplement: Supplementary Figure 3 — Association between the TIICs and clinicopathological features in RCC. (A) Infiltrating immune cells functioned in distinguishing the T stage. (B) Infiltrating immune cells functioned in distinguishing the M stage. [file Image_3.tif]

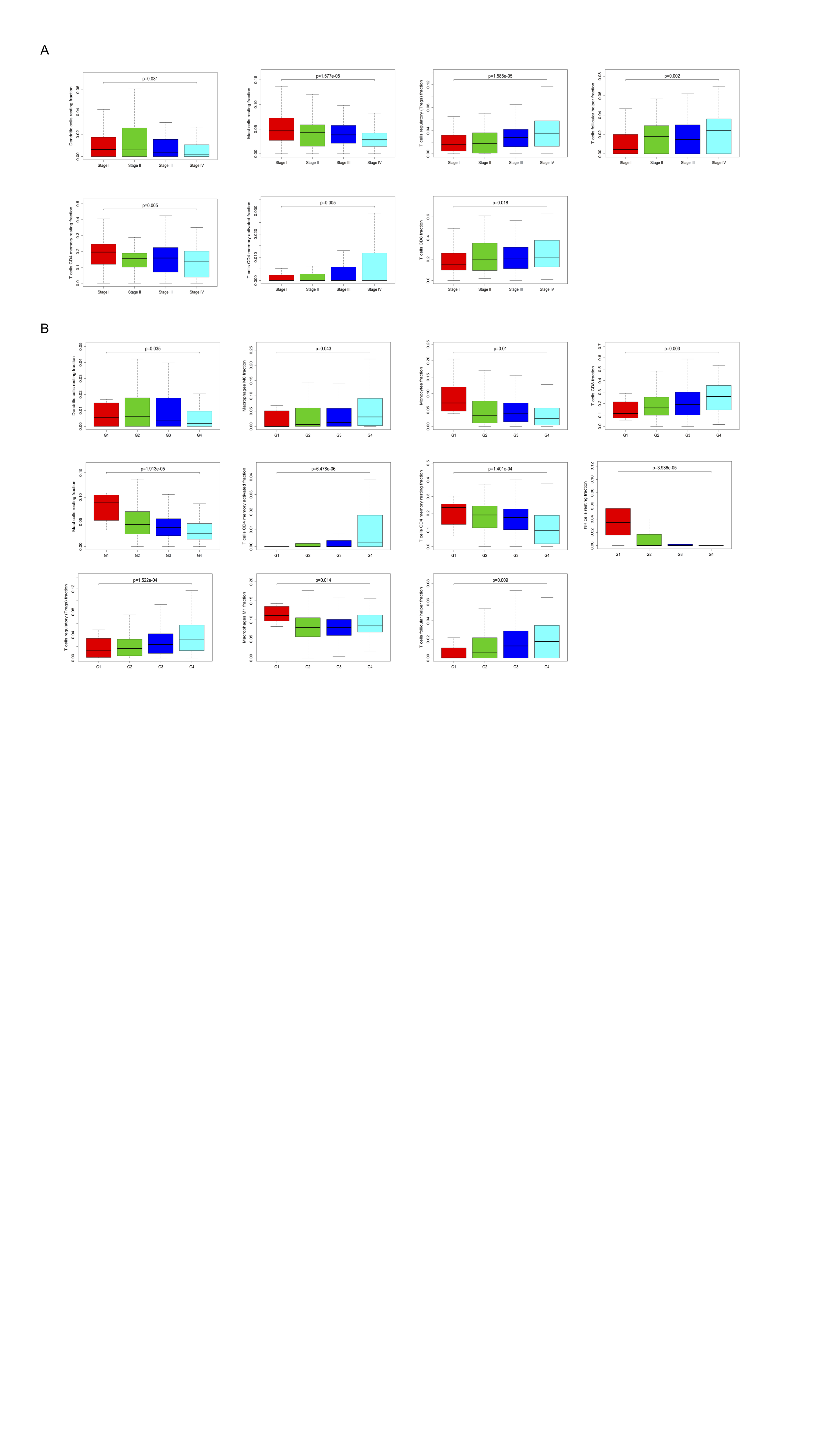

Supplement: Supplementary Figure 4 — Association between the TIICs and clinicopathological features in RCC. (A) Infiltrating immune cells functioned in distinguishing the pathological stage. (B) Infiltrating immune cells functioned in distinguishing grade. [file Image_4.tif]

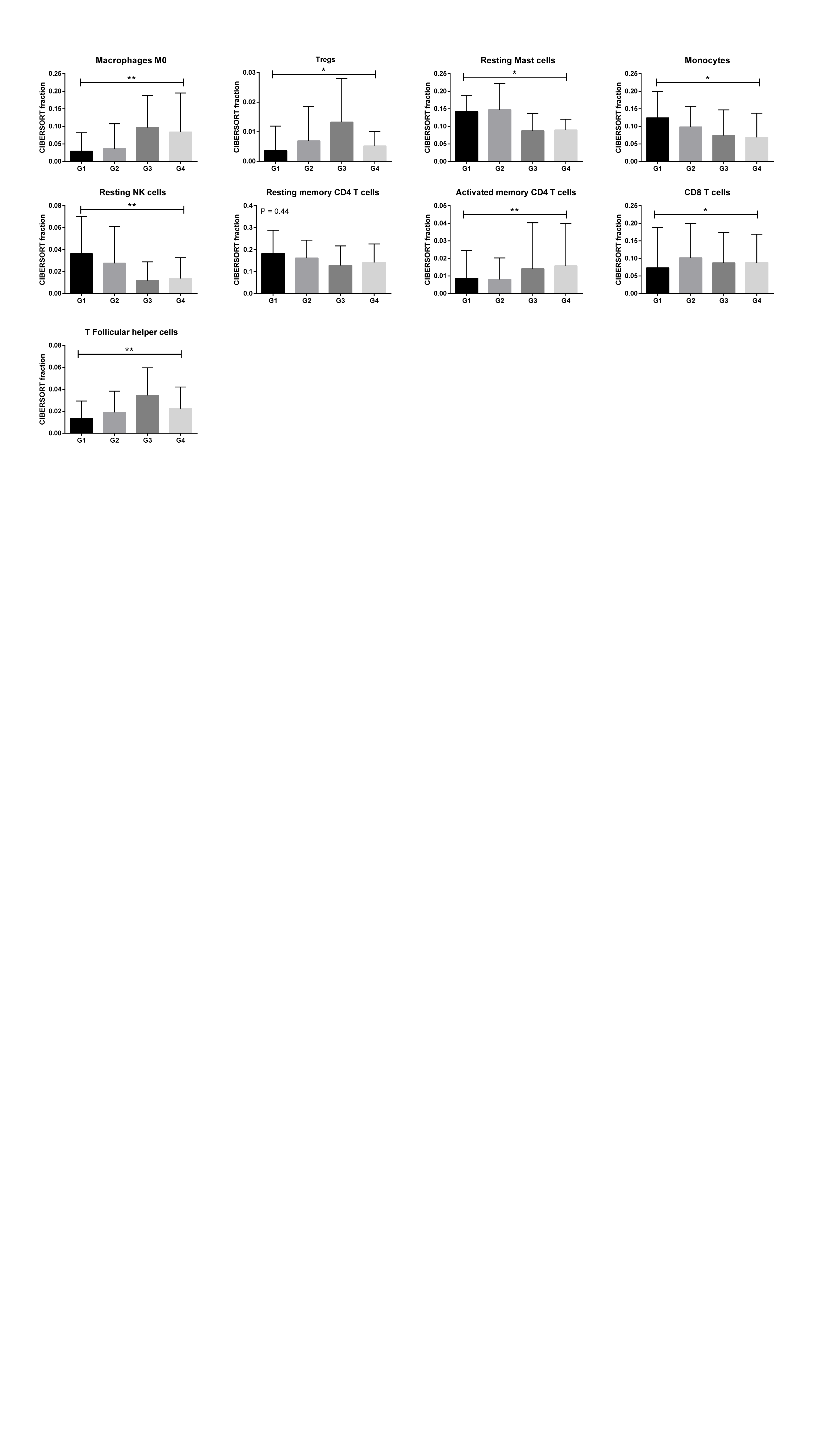

Supplement: Supplementary Figure 5 — Validation clinical characteristics of the TIICs in GSE40912. [file Image_5.tif]

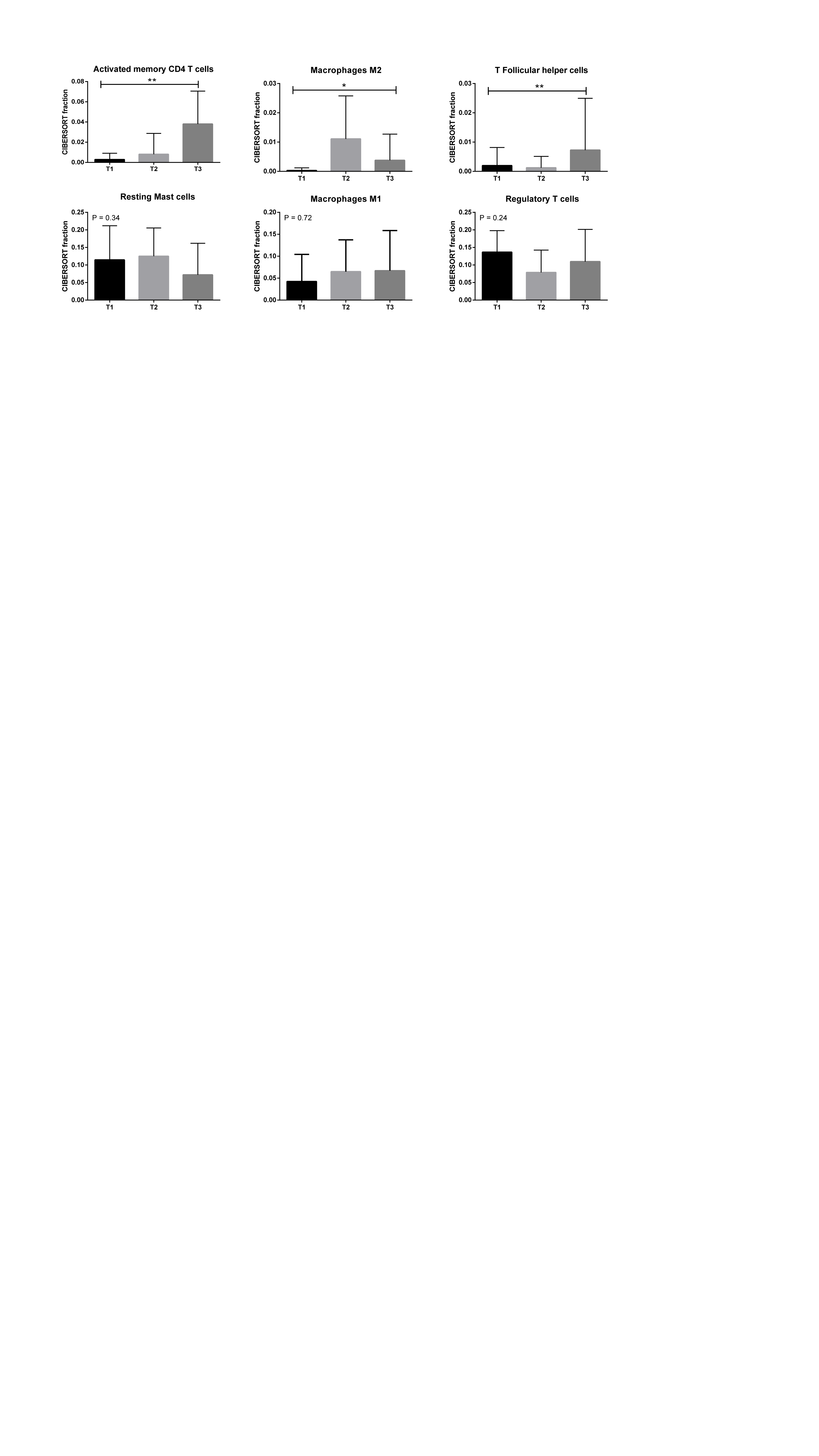

Supplement: Supplementary Figure 6 — Validation clinical characteristics of the TIICs in GSE79449. [file Image_6.tif]

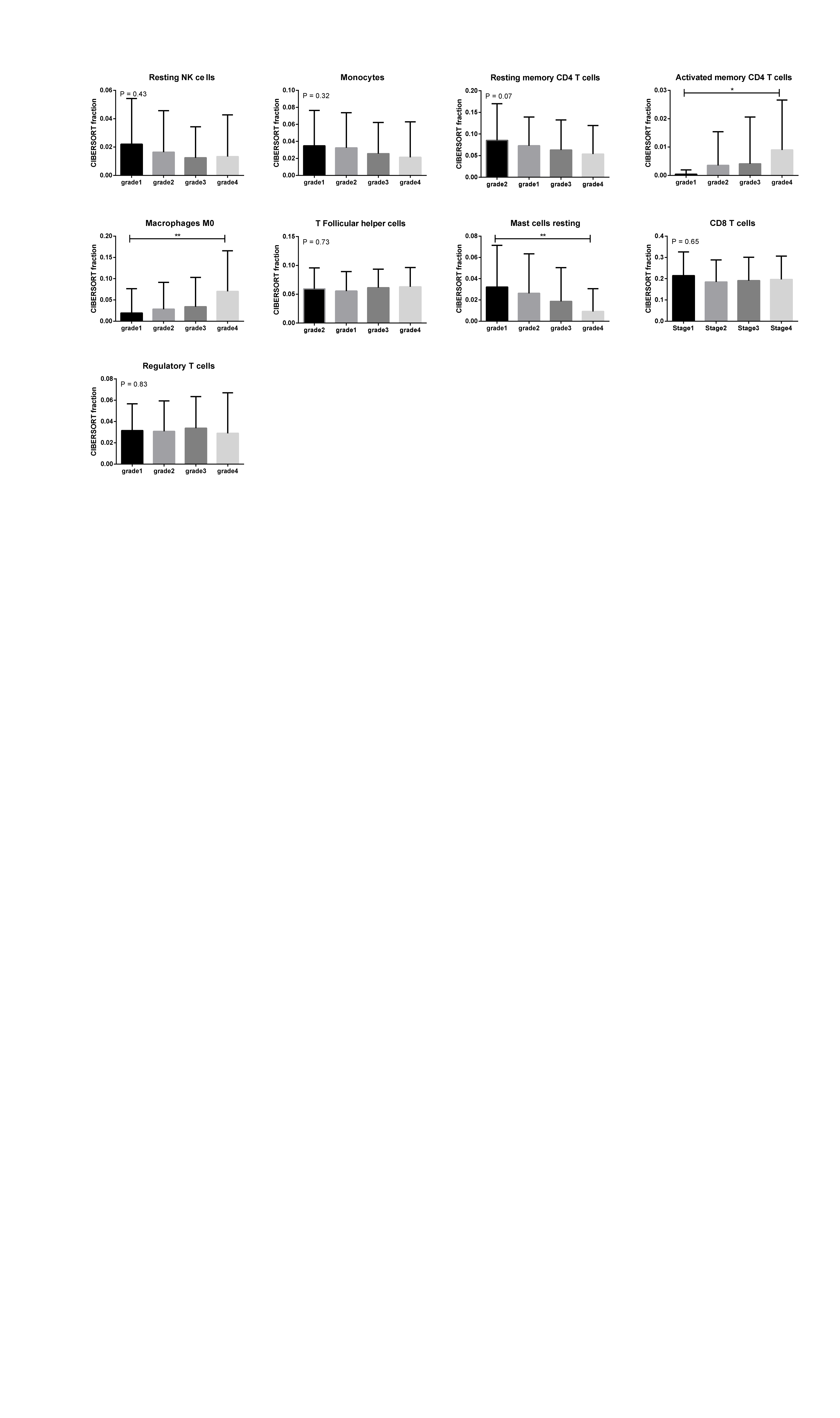

Supplement: Supplementary Figure 7 — Validation clinical characteristics of the TIICs in GSE68748. [file Image_7.tif]

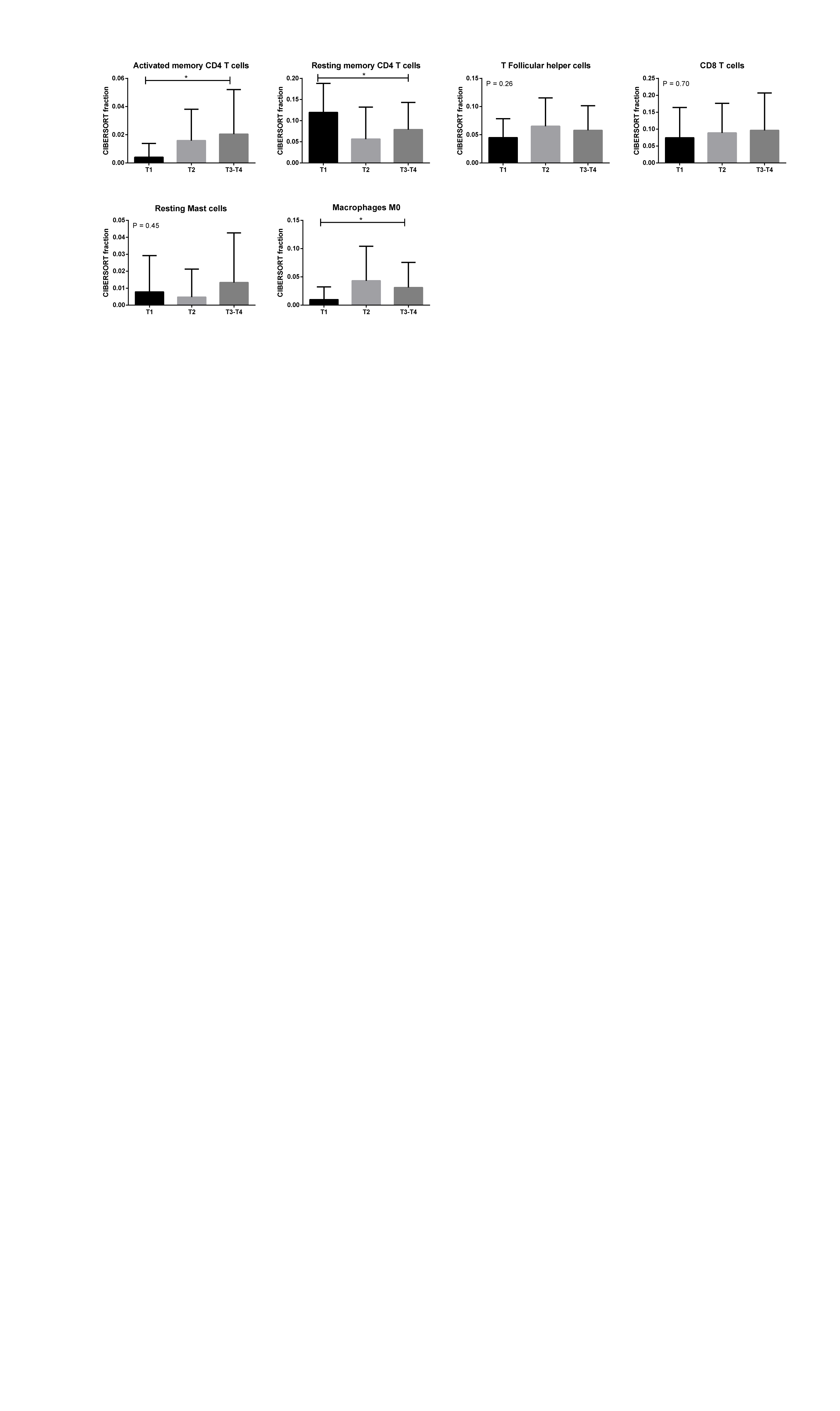

Supplement: Supplementary Figure 8 — Validation clinical characteristics of the TIICs in GSE73731. [file Image_8.tif]

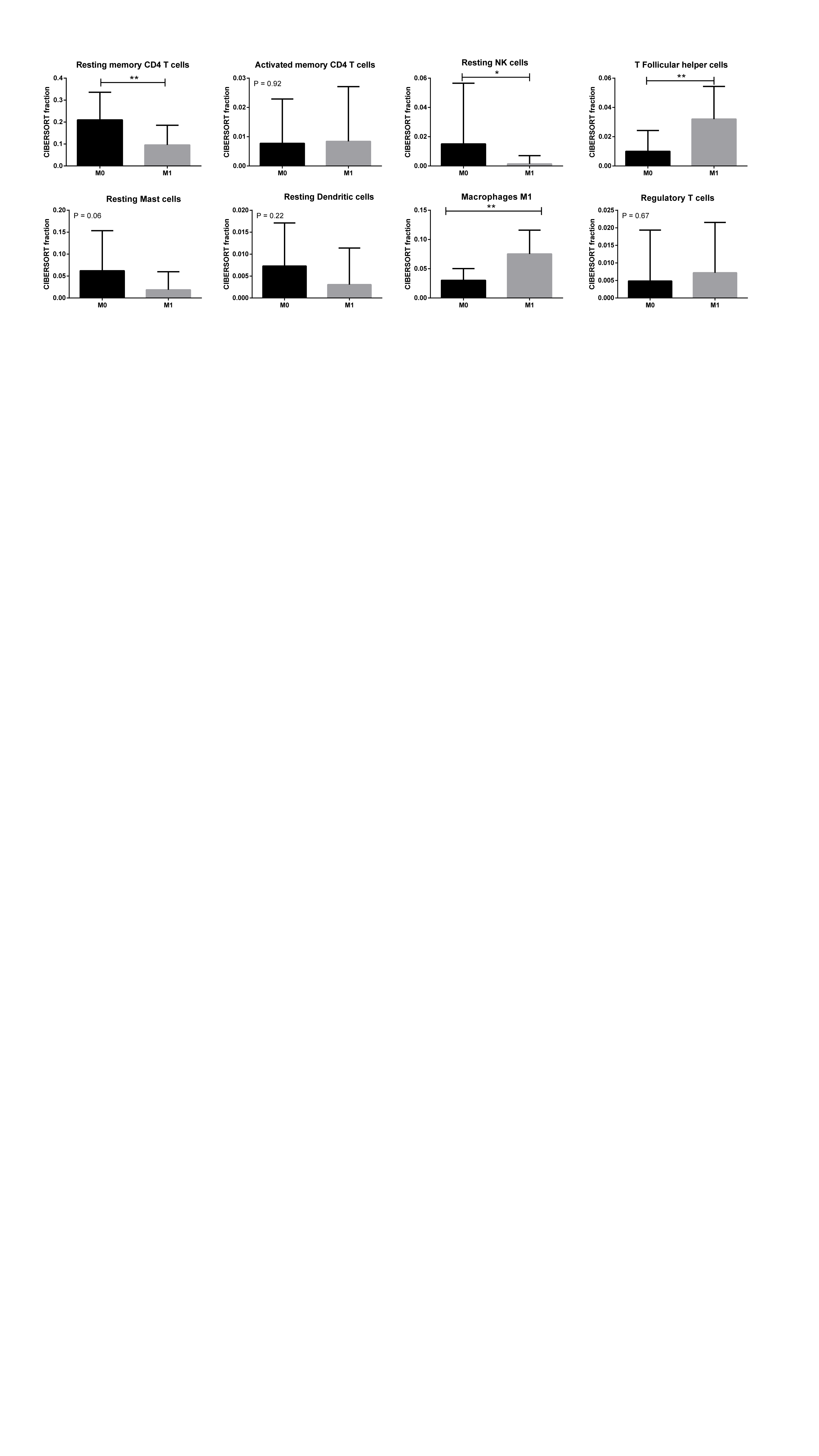

Supplement: Supplementary Figure 9 — Validation clinical characteristics of the TIICs in GSE40435. [file Image_9.tif]

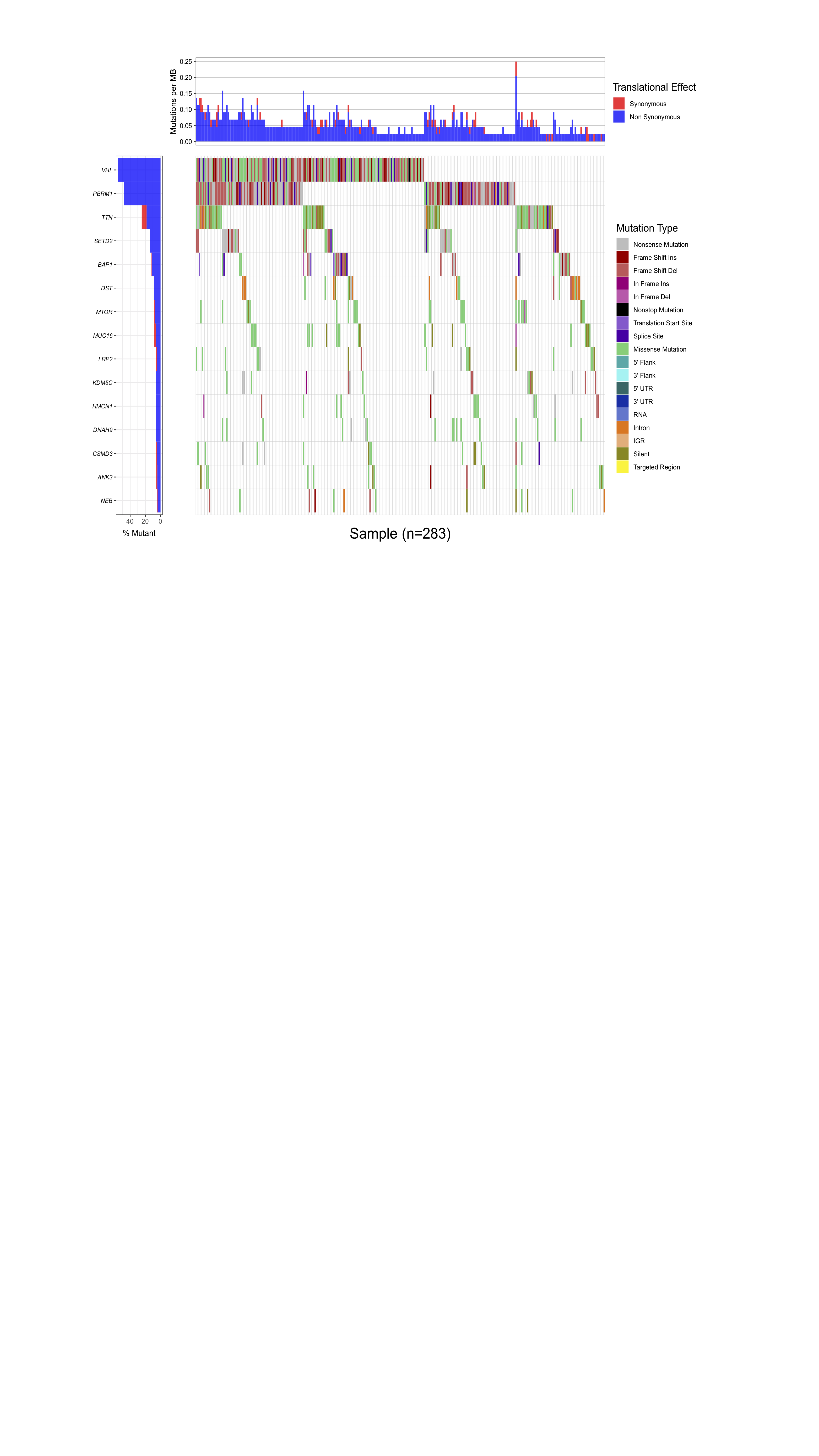

Supplement: Supplementary Figure 10 — Waterfall map depicts the top15 SNP in TCGA-KIRC. [file Image_10.tif]
