# Supplementary material for: Immune Infiltration Landscape in Clear Cell Renal Cell Carcinoma Implications
Source: Front Oncol. 2021 Feb 16;10:491621. doi: 10.3389/fonc.2020.491621 (PMC7923891; doi:10.3389/fonc.2020.491621)
Supplement: Supplementary file 11 [file DataSheet_1.docx]

Table S1 Specific gene markers for immune term

| **Immune responses** | **Gene** |
| --- | --- |
| Type I IFN Reponse | DDX4, IFIT1, IFIT3, IRF7, ISG20, MX1, MX2, RSAD2, TNFSF10 |
| Type II IFN Reponse | GPR146, SELP, AHR |
| APC co-inhibition | C10orf54, CD274, LGALS9, PDCD1LG2, PVRL3 |
| APC co-stimulation | CD40, CD58, CD70, ICOSLG, SLAMF1, THFSF14, TNFSF15, TNFSF18, TNFSF8, TNFSF9 |
| Check-point | IDO1, LAG3, TIM-3, IDO2, PDL-1, CTLA4, TIGIT |
| HLA | HLA-E, HLA-DPB2, HLA-C, HLA-J, HLA-DQB1, HLA-DQB2, HLA-DQA1, HLA-DQA2, HLA-A, HLA-DMA, HLA-DOB, HLA-DRB1, HLA-H, HLA-B, HLA-DRB5, HLA-DPB1, HLA-DRA, HLA-DRB6, HLA-L, HLA-F, HLA-G, HLA-DMB, HLA-DPA1 |
| MHC classI | B2M, HLA-A, TAP1 |
| Parainflammation | CXCL10, PLAT, CCND1, LGMN, PLAUR, AIM2, MMP7, ICAM1, MX2, CXCL9, ANXA1, TLR2, PLA2G2D, ITGA2, MX1, CD276, TIRAP, IL33, PTGES, TNFRSF12A, SCARB1, CD14, BLNK, IFIT3, RETNLB, IFIT2, ISG15, OAS2, REL, CD44, RRPAG, BST2, OAS1, NOX1, PLA2G2A, IFIT1, IFITM3, IL1RN |
| Inflammation-promoting | CCL5, CD19, CD8B, CXCL10, CXCL13, CXCL9, GNLY, GZMB, IFNG, IL12A, IL12B, IRF1, PRF1, STAR1,TBX21 |

Table S2 The clinical information of TCGA RCC patients

| Characteristics | No. of Patients (%) |
| --- | --- |
| Age (range) |  |
| Median (range) | 66 (30-90) |
| < 55 | 64 (15.9%) |
| ≥55 | 345 (84.1%) |
| Gender |  |
| Male | 265 ( 64.6%) |
| Female | 144 ( 35.4%) |
| T stage |  |
| T1-2 | 107 ( 26.2%) |
| T3-4 | 302( 73.8%) |
| Lymph node metastasis |  |
| No | 128 ( 31.2%) |
| Yes | 281 ( 68.8%) |
| Distant metastasis |  |
| No | 380 ( 92.9%) |
| Yes | 29 ( 7.1%) |
| TNM stage |  |
| Ⅰ-ⅠⅠ | 186 ( 45.5%) |
| ⅠⅠⅠ-Ⅳ | 223 ( 54.5%) |
| Status |  |
| Alive | 261 ( 63.7%) |
| Death | 148 ( 36.3%) |
| Follow-un (days) |  |
| Mean (range) | 493.78 (0 – 3720) |
